# Quality in Customer Service and Its Relationship with Satisfaction: An Innovation and Competitiveness Tool in Sport and Health Centers

**DOI:** 10.3390/ijerph16203942

**Published:** 2019-10-16

**Authors:** José Álvarez-García, Encarnación González-Vázquez, María de la Cruz Del Río-Rama, Amador Durán-Sánchez

**Affiliations:** 1 Financial Economy and Accounting Department, Faculty of Business, Finance and Tourism, University of Extremadura, 10071 Cáceres, Spain; pepealvarez@unex.es; 2 Business Management and Marketing Department, Faculty of Economics and Business, University of Vigo, 36310 Vigo, Spain; egzlez@uvigo.es; 3 Business Management and Marketing Department, Faculty of Business Sciences and Tourism, University of Vigo, 32004 Ourense, Spain; 4 Economy Department, Faculty of Economics and Business, University of Extremadura, 06071 Badajoz, Spain; amduransan@unex.es

**Keywords:** quality, perceived quality of the service, rating scale sports organizations (EPOD), sport and health centers, satisfaction

## Abstract

The objective of this research was to analyze the influence of the dimensions that enable the rating of service quality perceived by users of sport and health centers in the satisfaction they experience from the service received. In order to present the working hypothesis, a bibliographic review on the concept and dimensions of perceived service quality was carried out, as well as its relationship with satisfaction. The rating scale sports organizations (EPOD) was used as a measurement instrument. The application of a regression analysis was used to test the hypotheses. As a prior step, the measurement scales were validated and an exploratory factor analysis was applied to determine the structure of the variables considered. The regression models proposed show the joint influence of the dimensions used by the users to rate perceived service quality in their satisfaction. The results enabled us to observe that the dimensions considered in the model explained 75.7% of satisfaction, with the facilities and material, together with communication and activities, having the most significant influence on satisfaction. Meanwhile, dimensions that had less impact were the monitor and the staff. It is clear that there is a strong correlation between perceived quality and satisfaction with service.

## 1. Introduction

Currently, research shows that success and competitiveness in the management of sport and health centers requires more efficient management. In this sense, quality management, as one of the 25 most-used management tools [1], is essential [2]. Quality management is understood from two perspectives: The internal perspective (objective quality), focused on the standards of the service, and the external perspective (subjective quality), focused on quality as satisfaction of users’ expectations. The latter is currently the most-followed perspective in the service sector [3].

Thus, innovation and quality are the two key factors for business success [4,5,6,7,8,9]. Both concepts are linked in the sense that innovation is a part of continuous improvement [10] which, in turn, forms a fundamental part of quality. Porter [11] stated that the competitiveness of a country and, therefore, of its industrial and economic fabric, depends on the capacity to innovate and improve. With respect to organizations, innovation allows for economic sustainability and for their growth by generating competitive advantages [12,13,14,15,16].

Innovation is not exclusively associated with creativity and the generation of new products and services, but also refers to new forms of management and processes [17,18]. One of the most widely used definitions of innovation is provided by the Oslo Manual [19], which defines it as “the introduction of a new or significantly improved product (good or service), of a process, of a new organizational or marketing method, in the internal practices of the company, the organization of the workplace or external relationships”. Therefore, several types of innovation are distinguished: Product, process, organizational, and marketing innovation.

In this sense, the implementation of quality management systems is part of organizational innovation [20,21,22,23,24], since it involves the implementation of new organizational methods in the business. Therefore, the quality and, consequently, the implementation of quality management systems and the processes that are derived from it, become a tool for innovation and competitiveness in sport and health centers.

In the context of sports organizations, reference is made to service quality as “a differentiation strategy to increase productivity and profitability, as well as to improve the company’s image and achieve user loyalty” [25] (p. 250). In addition, service quality also enables knowledge of users’ perception of the quality of the service received, which is necessary to improve user satisfaction, as well as improve the competitiveness and viability of organizations. It should not be forgotten that satisfaction in the academic literature is considered a precedent for trust, mouth-to-ear communication [26], complaints [27], and loyalty [28].

In this research, carried out in the context of sport and health centers, the external perspective of quality is considered in which the client becomes the central axis of sports organizations. Therefore, it focuses on the concept of “perceived quality” of services, which is the way to conceptualize the predominant quality in the field of services.

In this sense, it must be taken into account that “a service is of quality to the extent that it meets or exceeds clients’ expectations” [29,30,31] and the concept is operationalized in practice by users comparing their expectations of the service with the perception that is formed once it is received [32]. In this way, quality ceases to be something objective (it focuses on the producer’s perspective) and instead becomes subjective, focusing on what the consumer says it is [29], as “only consumers judge quality: all other judgments are essentially irrelevant” [31] (p.18).

Research carried out on quality in sports services and consumer satisfaction has become important in recent years. According to Calabuig [33], it is mainly developed from three points of view in the sports sector: Psychosocial, the economic-business perspective, and the marketing perspective, focused on the consumer (studies based on SERVQUAL and alternative studies). This research follows the marketing perspective, whose research focuses, according to Pérez [34] (p.128), on “how to improve quality perception and the sense of satisfaction”.

Although several studies have been carried out following this perspective [35,36,37,38,39,40,41,42,43,44,45,46,47,48], authors such as Tsitskari et al. [49] and Arías-Ramos et al. [50] (p.106) state that these types of studies are not sufficient; “there are many issues to be addressed, lines of research to be continued and uncertainties to be resolved on the assessment of perceived quality and user satisfaction in sports organizations”.

In this context, this research is aimed at analyzing the influence of the dimensions that enable the rating of the quality of service perceived by users in the satisfaction they experience with it, which enables to us observe whether the perceived quality of a sports service is directly related to the satisfaction level. The empirical study was carried out in a sport and health center with a sample of 206 clients. The measurement instrument known as the rating scale sports organizations (EPOD) was used.

This article is structured as follows. After the introduction, where the subject matter of the study is contextualized, the study is justified and the objective is presented. Section 2 contains the theoretical reference framework (concept of perceived quality of the service and its relationship with satisfaction) and the work hypotheses are presented. The methodology used (target population, measurement questionnaire, and data analysis) is described below. The results are collected in Section 4, and finally, the conclusions obtained are discussed and presented.

## 2. Review of the Literature

### 2.1. Perceived Service Quality Concept

The starting point to define the concept of “perceived service quality” is defining the terms “service” and “service quality”. In this sense, the definition of service provided by Grönroos [30] is one of the first definitions and delimits service as that activity or series of activities of a more or less intangible nature that normally, but not necessarily, take place through interactions between the client and the employees of the service company who try to solve the consumer’s problems.

This definition, together with the one provided from a different approach by Lovelok, approach the perceived service quality concept by taking into account the satisfaction of expectations. Lovelok [51] (p.491) understands customer service as activities aimed at a task that includes interactions between clients and the organization and seeks the mutual satisfaction of the expectations of both, so it must be designed with two objectives in mind: Customer satisfaction and operational efficiency.

With regard to the service quality term, its definition is very complex since the intrinsic characteristics of the services means, on the one hand, that the quality practices applied must be different from those for tangible products [49,52] and, on the other hand, a greater difficulty is involved when evaluating the quality of a service. In this sense, Parasuraman et al. [32] (p.36) states that “the difference between the evaluation of the quality of a service and that of a good by a consumer is not in the process, but in the nature of the characteristics on which the evaluation is performed”.

These characteristics were specified by Parasuraman et al. [32]: Intangibility, inseparability of production and consumption, or simultaneity, heterogeneity, or variability, expiration. In Parasuraman et al. [32] and Grönroos [53], a broad discussion of the differences between services and physical goods can be seen. According to Zeithaml [54] and Stanton et al. [55], the intangible aspects are difficult to identify and quantify and make it difficult to establish precise specifications to standardize their quality. On the other hand, they are susceptible to different evaluations by clients, which makes the measurement and evaluation of quality difficult [55]. The inseparability in the services of production and consumption, as well as the perishable nature and the potential heterogeneity or variability in the performance, make the precision of quality difficult [32] (p.35).

In this context, as already mentioned, there are many definitions in this regard [54,56,57,58] and their review provides two different views or approaches when defining the service quality term: Objective and subjective quality [53] (p.38). On the one hand, the objective quality or internal vision of quality focuses on the technical aspects [59] from the producer’s perspective, as well as the subjective quality or external vision of quality in which clients’ requirements are emphasized, thus emerging the “perceived quality” concept. This last concept was introduced by Gönroos [58] when considering the idea that clients compare their expectations with the service received, with the result of this process being the perceived quality of the service. This concept was developed later, both methodologically and empirically, by Parasuraman et al. [32,60]. An important aspect to mention is that these two visions gave rise to two schools of thought: The Nordic School and the North American School.

In the case of the Nordic School, its main representatives Grönroos [61,62,63], Gummesson [64], and Lehtinen and Lehtinen [65], focus on the concept of service quality from the point of view of the product, with efficiency being the basic objective for which standards are used for its control [65]. The Grönroos Service Quality Model [58] established two dimensions for service quality which interact between each other: Technical quality or design of the service, referred to as “what” service the client receives (result), being susceptible to be measured by the company and evaluated by the consumer; and the functional quality or performance of the service, which deals with “how” customer service (process) is provided. Both dimensions are compared with previous expectations by the client, which are influenced by the result of the service, by the way it is received, and by the corporate image [58]. Subsequently, this conceptual model of Grönroos, in which perceived quality is defined as a result of the comparison between the expected and received service, was moved to the United States and developed by Parasuraman, giving rise to the emergence of the North American School.

Bearing in mind that this last perspective is the one that best fits sports services, which is the scope of study in this research, it is the one that was developed in more detail. Thus, Parasuraman et al. [66] (p.3) defined perceived service quality by the client, as an overall assessment of the consumer regarding the superiority of the service resulting from the comparison made by clients between the expectations and perceptions regarding the performance of the service received. This definition of perceived quality became the most widely used way to conceptualize quality from the perspective of services and is the basis of the theoretical and methodological approach of Parasuraman et al. [32], in which the quality process in services is explained.

These authors posed the question of “What is service quality?” in their initial investigation. Thus, the concept of perceived quality [32] arised. They also determined the dimensions used by clients to rate services [66]. Finally, they developed a conceptual and empirical model to measure service quality: The SERVQUAL model, represented graphically by Zeithaml et al. [67] (p.26), and defined as “a summarized multiple-scale tool with a high level of reliability and validity that companies can use to better understand the expectations and perceptions that customers have regarding the service received”.

As shown, the two factors that determine perceived service quality are expectations and perceptions [66]. Expectations are defined by Parasuraman et al. [66] (p.17) as clients’ desires or needs and they are determined, as reflected in the conceptual model, by previous experiences, clients’ current needs and demands, the company’s external or formal communications, mouth-ear communication between clients, and the corporate image. Perceptions are defined as consumers’ beliefs regarding the service received, which will be determined by the dimensions which clients consider in order to rate the service.

In this regard, in the literature on the subject, there are divergences regarding these dimensions and there is no consensus. Garvin [68] consider eight dimensions (performance, characteristics, reliability, attachment, durability, service aspects, aesthetics, perceived quality), Lehtinen and Lehtinen [65] consider three dimensions (physical, corporate, and interactive quality), and Grönroos [58] takes into account the technical or result dimension, the functional or process dimension, and the corporate image.

However, the most-considered multidimensionality of service quality by researchers in this field is the one proposed by Parasuraman et al. [32], who consider that perceived quality is made up of 10 dimensions: Tangible elements, “appearance of physical facilities, equipment, staff and communication materials”; reliability, “ability to implement the service promised reliably and carefully”; responsiveness, “willingness to help customers and provide them with a quick service”; professionalism, “having the required skills and knowledge of the process of providing the service”; courtesy, “attention, consideration, respect and helpfulness of the contact staff”; security, “no dangers, risks or doubts”; credibility, “veracity, belief, honesty in the service provided”; accessibility, “accessible and easy to contact”; communication, “keeping clients informed using a language they can understand, as well as listening to them”; and understanding the client, “making the effort to know the clients and their needs” [67] (p.24). Subsequent research by these authors [66] reduced them to five dimensions: Tangible elements, reliability, responsiveness, security (including professionalism, courtesy, credibility, and security), and empathy (including accessibility, communication, and understanding of the user).

In summary, the concept of perceived service quality is a complex variable, with several definitions in this regard. This was observed by Díaz and Pons [69] (p.53), who, after analyzing the literature on perceived service quality, proposed two perspectives when defining the concept: From the perspective of customer perception [54,70,71] and from the perspective of customer expectations and perceptions [32,72,73]. However, in recent years, the most recurring perceived quality concept has been one which contextualizes quality in the field of services from the client’s perspective and is conceptualized by comparing the client’s expectations with the perceptions about the service received. According to Zeithaml et al. [31] (p.18), “only consumers judge quality: all other judgments are essentially irrelevant”.

The research conducted by Grönroos [58] and Parasuraman et al. [32,66], aimed at defining the concept of perceived quality, gave rise to two schools and their corresponding models of perceived service quality. As Gómez [74] (p.53) states, “to have a more complete vision and to finish understanding the concept of perceived service quality, it is necessary to know the different theoretical models based on this construct”. In the case of the North European or Nordic School, its integral models are the following: Models of quality of service or image [58], the Quality Model of Grönroos and Gummerson [53], augmented service offering [53], “Servuction” Model by Eiglier and Laneard [75], and the three-component model [76]. The North American School integrates six models: The SERVQUAL Model [32], Augmented Quality Service Model [77], SERVPERF Model [78], Multidimensional, Hierarchical Model [79], service quality model of Bolton and Drew [80], and Bitner service quality model [81].

### 2.2. Relationship between Service Quality and Consumer Satisfaction

In the previous section, the concept of perceived quality was broadly discussed, so the starting point of this section is to define the term “consumer satisfaction”. Two major lines of research in recent years, the cognitive model [82] and emotional model [83], have been integrated, leading us to consider satisfaction as a post-consumer response or assessment [84] susceptible to change in each transaction [85].

There is a great similarity between the concepts of perceived quality and satisfaction [86]. However, most researchers suggest that both concepts are different constructs and that service quality is a broader concept than satisfaction. Thus, Parasuraman et al. [66] refer to the differences between both concepts in relation to durability. Thus, perceived quality refers to an enduring attitude related to the superiority of a service, while satisfaction is a transitional assessment of a specific transaction in which a comparison is made with what was expected [85]. To Oliver [87], the differences are that when the consumer assesses the perceived quality, the predominant dimensions are those of a cognitive nature and, in the case of satisfaction, they are emotional in nature.

These differential characteristics, which are discussed in the literature, led Zeithaml et al. [86] to propose that the difference between both concepts is based on the fact that satisfaction involves an assessment made by the client for a specific transaction [88] and requires previous experience, since this assessment depends on the consumer’s previous expectations [76,89], whereas service quality can be perceived without the need for a direct experience with it [66].

There are many who affirm the existence of a relationship between both concepts [54,66,78,90]. However, they do not reach a consensus regarding the causal relationship between both concepts. Thus, Iacobucci et al. [91] state that there are two clearly differentiated positions: Those that support the idea that satisfaction is a consequence of perceived quality [66,78,92,93,94,95] and research that supports the inverse relationship, considering satisfaction as an antecedent of service quality [59,80,81,96,97]. However, there is also an intermediate position, in which satisfaction is considered both an antecedent and a consequence of the perceived quality of service. Representatives of this position are Parasuraman, Zeithaml and Berry [98], Rust and Oliver [76], and Martínez-Tur, Peiró and Ramos [85].

In this context, the following working hypotheses were proposed:

H1: The service quality dimensions have a positive influence on the satisfaction experienced by the users of sport and health centers.

H2: The service quality dimensions have a positive influence on the satisfaction with the facilities experienced by the users of sport and health centers.

H3: The service quality dimensions have a positive influence on the satisfaction with the organization of activities that the users of the sport and health centers experience.

H4: The service quality dimensions have a positive influence on the satisfaction with the activities experienced by the users of sport and health centers.

## 3. Methodology

### 3.1. Universe, Sample, and Questionnaire

The research was designed by organizing the collection of data in order to comply with the proposed objective through a structured questionnaire addressed to users of a sport and health center. To calculate the representativeness of the sample, only the subscribers were taken into account. Users who use the service occasionally, which represent a very small percentage, were excluded. Thus, the target population was made up of 1512 subscribers, and 206 users responded to the questionnaire (incomplete questionnaires were discarded), which represents a response rate of 13.62% and a margin of error of 6.35%, taking into account a 95% confidence level (Z = 1.96 p = q = 5).

The questionnaire was structured in three parts. First, to measure the perceived service quality, the rating scale sports organizations (EPOD) was created by Nuviala et al. [99], adapted to the sport and health centers where the study was carried out (29 items). This scale “is a tool for practical and direct application on the perception that users of sports services have of the sports organization and the services it provides” [99] (p.10). The original scale consists of 28 items grouped into four dimensions: Sports experts, facilities and material resources, activities, and image of the organization.

The second part of the questionnaire included the scale to measure user satisfaction with the service, which was divided into three dimensions: Satisfaction with the facilities (five items [100]) satisfaction with the organization (three items [99]), and satisfaction with the development of the activity (four items [101]). The last part of the questionnaire included the data that enabled us to define the sample profile. Five-point Likert measurement scales were used (1–totally disagree to 5–totally agree and 1–not at all satisfied to 5–very satisfied).

Regarding the profile of the user of the sport and health center, the user is between 18- and 40-years-old (77.6%), male (66.99%), student occupation (32.4%), or works in the private sector (31.07%), with a secondary education level (49.51%). This user usually attends the sport and health center three days a week (43.69%) on average, preferably in the afternoon (55.83%). The reasons for being a user of the center are: Proximity to home or work (20.55%), because of the treatment received (10.84%), and because of the range of activities desired (9.22%). The main reason for sports is entertainment in 40.29% of cases and for aesthetic reasons in 31.07% of cases.

### 3.2. Data Analysis Techniques

The statistical program SPSS 19.9 (IBM, Armonk, NY, USA) (Statistical Package for the Social Sciences) was used to perform the data analysis and was carried out in two phases. First, a descriptive study of the sample (mean and standard deviation) was carried out and the measurement scales were validated, taking into account the psychometric properties of reliability, validity, and unidimensionality [102]. To evaluate the reliability and delimit the number of items that measure each scale, Pearson’s item-total correlation coefficients were considered (they should not exceed 0.3 according to Nunnally [103]) and Cronbach’s α [104] was estimated (must be greater than 0.7).

The analysis of the unidimensionality enabled to us to find the structure of dimensions of the proposed scales. Prior to its performance, it was found that the data were adequate to perform the exploratory factor analyses: Analysis of the correlation matrix, Bartlett’s Sphericity test (χ^2^ high and sig. > 0.05), the Kaiser-Meyer-Oklim (KMO) measure (>0.7, median: >0.8, good and 1> = KMO > 0.9, very good), and the sample adequacy measure were acceptable (unacceptable for values lower than 0.5, small values should be removed from the analysis). Unidimensionality was tested through the percentage of variance explained and the factor loadings of each indicator, for which an exploratory factor analysis of main components with varimax rotation was carried out [105].

Second, the multiple regression analysis was applied to contrast the hypotheses proposed. This process enabled us to study the relationship between a dependent variable (satisfaction) and its independent or predictive variables (dimensions of perceived service quality) through the estimation of the regression coefficients that determine the effect that the variations of the independent variables have on the behavior of the dependent variable. Prior to the regression analysis, the underlying assumptions on which this type of analysis is based were verified (linearity, independence, homoscedasticity, normality, and noncollaterality).

## 4. Results

### 4.1. Validation of Measurement Scales

First, the internal consistency of the scale that measures the perceived quality of the service was analyzed through reliability analysis (item-total correlation and Cronbach’s α). Taking into account the item-total correlation, it was not necessary to eliminate any items, since all of them were above the recommended minimum of 0.3. Cronbach’s α that measures the reliability of each factor is higher than the recommended minimum 0.7 [103].

In order to analyze the unidimensionality of the scales, an exploratory factor analysis was carried out, which enabled us to group the items and identify five factors or dimensions that explain 70.28% of the total variance (it exceeds the minimum requirement of 50%) (Table 1). The application of this analysis involved the elimination of the item “with this activity I obtain the results I expected” since the factor loading was less than 0.5 [106].

In the case of the satisfaction scale, the factor analysis resulted in three factors that were denominated (“satisfaction with the facilities”, “satisfaction with the organization of activities”, and “satisfaction with the activity”), which explain 73.77% of the total variance (Table 2). The analysis of the item-total correlation did not involve eliminating any items, since they were higher than 0.3 in all cases.

From the results of the analyses carried out to corroborate reliability once the item “with this activity I obtain the results I expected” was eliminated in the scale that measures the perceived quality of the service, it can be concluded that the proposed scales are highly reliable, thus being free of random errors, and are able to provide consistent results.

### 4.2. Regression Analysis

Four multiple regression analyses were proposed in order to corroborate the objectives set. The models included six independent/predictive variables that corresponded to the dimensions included in the scale, enabling us measure the perceived quality of the service (monitor, facilities and material, activities, communication, and staff) with each of the dimensions of satisfaction that have been considered (dependent variable or criterion variable): Overall satisfaction, satisfaction with the facilities, satisfaction with the organization of activities, and satisfaction with the activities. Two control variables were incorporated into the model: Users’ sex and age.

First, the results obtained between the analysis variables in the correlation matrix were analyzed (Table 3). Regarding the control variables, although no significant differences were found and the correlation coefficients are weak, it is observed that the age variable negatively affects the satisfaction with the facilities and with the organization and positively affects the satisfaction with the activities and overall satisfaction. The correlation coefficients allow us to affirm that the dimensions of the perceived quality of the service have a positive relationship with satisfaction (H1, H2, H3, H4), with strong and significant correlation coefficients at the *p* < 0.01 level.

Prior to the regression analysis, the underlying assumptions on which this type of analysis is based were verified (linearity, independence, homoscedasticity, normality, and noncollaterallity). For the assumption of independence of residuals, the Durbin-Watson statistics was obtained, which, in the three regression models built, gave values between 1.5 and 2.5 (Table 2). In all cases, it gave values lower than 2, which indicates positive autocorrelation.

**Figure 1:**
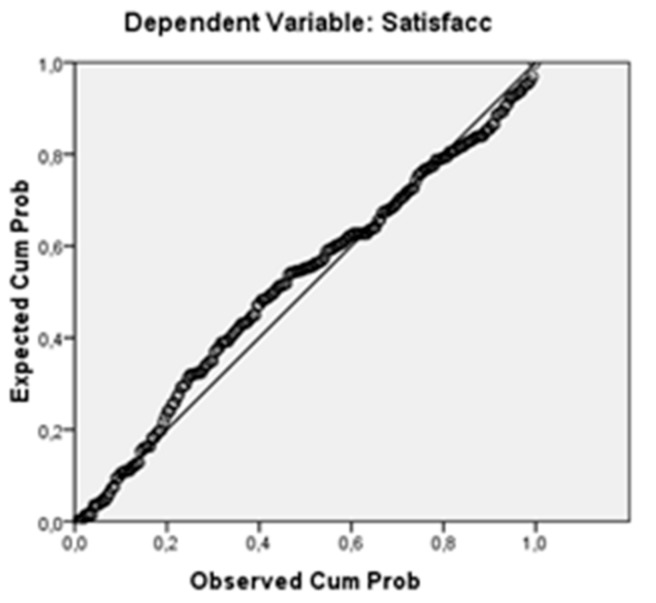
Standardized waste. Source: Authors’ own data

In the case of collinearity, its diagnosis provided tolerance values between 0.302 and 0.556, which indicate noncollinearity. Therefore, none of the independent variables have correlations greater than 0.9. Moreover, it is possible to assume residual normality, since this tendency could be verified in the analysis of histograms and, in addition, it was confirmed by calculating the Kolmogorov-Smirnov test. Finally, regarding the homoscedasticity assumption, for each value of the independent variables in the scatterplot (Figure 1), the residuals are distributed in a similar way (no relationship was observed between the forecasted values and the residuals).

In the regression analyses carried out, the measure of the goodness of fit of the model was estimated using the multiple correlation coefficient and the coefficient of determination, which is the square of the previous multiple correlation efficient and expresses the proportion of the variance of the dependent variable explained by the regression model. It is observed that the proposed models have an adequate goodness of fit. In this sense, the explanatory variables contained in the model explain 75.7% of overall satisfaction, 53.4% of the satisfaction with the facilities, 56.1% of the satisfaction with the organization of activities, and 56.0% of the satisfaction with the activity (Table 4). In addition, the F statistic, which enables us to decide whether there is a significant relationship between the dependent variable and the set of independent variables taken together, provides a good adjustment to the point cloud (sig 0.000, indicates that there is a significant linear relationship).

Second, the partial correlation coefficient of each explanatory variable was estimated, which indicates the specific relationship of the variable with the dependent variable assuming that the other independent variables remain constant. The sign of the correlation coefficient β makes it possible to determine the direction of the relationship and the F statistic, as well as the goodness of fit of the regression. The *p*-value (> or < that 1) indicates the significance level with the dependent variable.

The results obtained in the regression together with the correlations enable us to observe that in the case of “general satisfaction”, the dimensions of perceived service quality contribute significantly to explain satisfaction with high and significant β values at a *p* < 0.001 level. All dimensions, except for the monitor dimension (*p* < 0.05), explain satisfaction at a *p* < 0.001 level. In this sense, the higher the perceived quality of each of the dimensions, the higher the satisfaction experienced by the users.

In this same line, the other three models of multiple regression were proposed with the objective of identifying which dimensions of perceived quality affect the satisfaction that users experience regarding the facilities, organization, and the activities, and to what extent. In the case of satisfaction with the facilities, it is observed that the monitor, the activities, and the staff do not contribute significantly to explain satisfaction (sig > 0.05). As expected, communication significantly contributes to explain satisfaction (β = 0.160, *p* < 0.05), along with the facilities dimension (β = 0.540, *p* < 0.001).

The regression model, which explains the satisfaction with the organization of activities, is significantly influenced by the organization of activities (β = 0.399, *p* < 0.001), staff (β = 0.180, *p* < 0.001), and communication (β = 0.149, *p* < 0.001), while the relationship with the facilities and the monitor is not significant (sig > 0.05). Finally, in the last model, which refers to satisfaction with the activities, all the variables are significant at the *p* < 0.001 level (communication and staff) or *p* < 0.05 level (monitor and facilities) except for the activities dimension.

Taking the results into account, the hypothesis H1 and, partially, H2, H3, and H4, are corroborated, since not all dimensions positively and significantly influenced satisfaction.

## 5. Discussion 

First, note the results obtained related to the dimension structure of the scale, which enables us to measure the perceived quality of the service. In this research, the rating scale sports organizations (EPOD), developed by Nuviala et al. [99], was used. Since it was applied to a sample of users of an organization that provides sports services, but with different characteristics from the sample of the original scale, its reliability and unidimensionality were analyzed and studied.

In the case of reliability, Cronbach’s α, which measures the reliability for the total scale, is 0.962, which is very similar to that obtained by Nuviala et al. [99] of 0.916. If each one of the dimensions is taken into account, it is higher than 0.8 in all cases, corroborating the results obtained by Nuviala et al. [99] that obtained values between 0.799 and 0.885. Therefore, it was concluded that the scale is reliable.

Regarding the structure of dimensions in this investigation, the items were grouped into six dimensions: Monitor (six items), facilities (five items), sports equipment (four items), activities (nine items), communication (three items), and staff (three items). However, in research by Nuviala et al. [99], items were grouped into five dimensions (activities, sports experts, spaces, materials, image). The scale measuring satisfaction was divided into three factors or dimensions: Satisfaction with the facilities, with the organization, and with the activity, which correspond to the three scales proposed by Wicker et al. [100], Nuvialia et al. [99], and Graupera et al. [101], and which refer to satisfaction with three aspects or different areas of the sports center, corroborating its reliability.

Once the structure of the considered scales was discussed, the results obtained in this research were discussed relating to the four hypotheses that enabled us to observe the relationships with the quality they perceive and their satisfaction. According to the results, Hypothesis H1 was corroborated, which considered the positive relationship between the dimensions of perceived service quality and overall satisfaction (H1). It was confirmed that the relationship exists (in all cases, the standardized correlation coefficients are significant at the *p* < 0.001 level). These results, in the case of the relationship with overall satisfaction, are corroborated by empirical studies conducted by Bisschoff and Lotriet [107], Kyle et al. [108], Murray and Howat [94], Shonk and Chelladurai [109], and Nuviala et al. [110], which state that a greater level of quality service perception results in greater satisfaction.

Finally, in order to reinforce the validity of the hypotheses and study the relationship structure, different regression models were proposed, which included the perceived service quality dimensions as independent variables and overall satisfaction, satisfaction with the facilities, with the organization, and with the activities as dependent variables, with the purpose of evaluating the joint effects of the independent variables on satisfaction.

The first model showed that the hypothesis H1 was corroborated. All the dimensions of the perceived quality scale influenced satisfaction positively and significantly (on overall satisfaction, *p* < 0.001), with the variables included in the model explaining 75.7% of satisfaction. This clearly shows that the quality dimensions are closely related to satisfaction, with the most influential variables being facilities and material (β = 0.325, *p* < 0.001), followed by communication and activity (β = 0.225 and 0.214; *p* < 0.001). The least influential variables were to HR, monitor, and staff (β = 0.105 and 0.162, *p* < 0.001). Studies of a quantitative nature corroborate this result. For example, the study carried out by Nuviala et al. [110], included the perceived value, with 55.6% of satisfaction explained by their model, in addition to the perceived quality dimensions. On the other hand, the dimensions “activities and sports experts” were the most relevant in the regression equation, with β values of 0.347 and 0.266, respectively, with the “value and material” variables being the least important, with a β value of 0.074. These results differ from those obtained in this study. On the contrary, other in the study conducted by Mañas et al. [44], as well as in this study, it was found that tangible elements are important predictors of user satisfaction.

On the other hand, hypotheses H2, H3, and H4 were partially corroborated. It was observed that in the case of satisfaction with the facilities (H2), the facilities dimension (β = 0.540, *p* < 0.001) together with communication (β = 0.160, *p* < 0.05) were the only two dimensions that influenced satisfaction and explained 53.4%. These results show that a center interested in in improving its users’ satisfaction with its facilities must comply with the requirements and expectations of its clients regarding cleanliness, safety, temperature, and sports equipment. In addition, it should pay special attention to the communication mechanisms implemented in its organization. In this sense, the client expects the center to have a procedure of complaints and suggestions, and all those channels necessary to achieve adequate communication with its users.

If the regression model explaining satisfaction with the organization of activities is observed, the independent variables that positively and significantly influenced satisfaction were activities (organization and development), staff, and communication. The users did not consider the dimensions monitor and facilities and material when forming their satisfaction. In the case of satisfaction with the activities, four of the five dimensions influenced satisfaction to a greater or lesser extent, with the exception of the activity dimension, which was not significant (β = 0.095, *p* > 0.05), while the communication dimension was the most important. The comparison of these results with other studies is complex due to the differences between the measurement instruments used and the dimensions evaluated.

In short, it is observed that the dimensions of perceived quality related to HR (monitor and staff) were the least influential in the satisfaction experienced by the users of the sport and health center, with the dimension facilities and material the most important together with activities and communication, which show a very similar influence. The comparison of these results with other studies is complex due to the differences between the measurements used and the dimensions evaluated.

## 6. Conclusions

Before starting the presentation of the conclusions, note that this research work is novel since it aims, on the one hand, to fill a gap in research carried out in the sports organizations sector. In this sense, the relationship between perceived service quality and satisfaction has been studied extensively, and this has been corroborated [94,107,108,109]. However, there is still no consensus on the causality of the relationship, so it is necessary to continue conducting research in this regard [91].

There are many investigations that develop measurement scales of perceived quality. However, there are very few investigations that analyze which quality dimensions are the most important to form the client’s satisfaction. This research takes into account what was discussed by Szabó [111] and Tsitskari et al. [49], who state that the study of quality in the sports industry is in its early stages, so it is essential to continue doing research and deepening knowledge in this area [111]. In this sense, this research allowed the analysis and reinforcement of some of the conclusions already obtained in other studies.

The results of this investigation have significant academic implications and are of great interest to organizations that provide sports services, enabling the observation of how they jointly affect the dimensions of perceived service quality in the formation of their users’ satisfaction, becoming a key strategic element for any organization. In this sense, it is important to bear in mind that sport and health centers, like any other organization, must improve the quality of the services they provide in order to satisfy their users, and it is necessary to listen to users and measure their satisfaction. This will enable these organizations to adjust their service to the existing demand and to anticipate and adapt to the changes in users’ tastes since, as stated by Súarez et al. [112] (p. 30), “who determines the quality of a service is the user through his/her satisfaction”.

The first limitation of this study that the study was carried out in a single sport and health center. In future research, the studies should be extended to other sport and health centers, so the results should be extrapolated with caution. Another limitation of this study is its cross section.

## Figures and Tables

**Table 1:** Perceived service quality: Descriptive statistics and exploratory factor analysis.

Exploratory Factor Analysis ^1^: Cronbach’s ∝ = 0.962; χ^2^ (sig.): 5090.804 (0.000); KMO: 0.939; Measure of Simple Adequacy (MSA): (0.916−0.900) % Variance: 70.28	Item-Total Correlation	Mean *	S.D. ^2^	Loadings
**Factor 1: monitor/coach (Mean = 4.41; Eigenvalue = 6.100; %Variance = 20.33; Cronbach’s ∝ = 0.937)**				
The monitor is respectful with the timetable	0.673	4.33	0.74	0.780
You are happy about how you are treated by the monitor	0.650	4.37	0.75	0.824
You believe that the monitor has provided adequate attention to the users since the first day	0.638	4.36	0.72	0.815
You believe that the monitor adapts the classes to the users’ interests/needs	0.603	4.41	0.70	0.833
You consider that the monitor encourages the group sufficiently	0.568	4.47	0.70	0.806
You perceive that the monitor has well-planned classes	0.677	4.47	0.72	0.774
**Factor 2: facilities and material (Mean = 4.39; Eigenvalue = 5.205; % Variance = 17.35; Cronbach’s ∝ = 0.932)**				
The changing rooms are clean enough	0.685	4.41	0.75	0.577
The changing rooms are large enough	0.666	4.50	0.68	0.589
The facilities are clean enough	0.750	4.42	0.82	0.767
The temperature is adequate	0.713	4.38	0.84	0.782
The security of the facilities is adequate	0.725	4.50	0.76	0.774
Sufficient material is available for classes	0.701	4.31	0.75	0.567
The material is in optimal conditions for its use	0.678	4.25	0.90	0.752
The material is modern	0.700	4.30	0.93	0.635
**Factor 3: activities (Mean = 4.41; Eigenvalue = 3.679; % Variance = 12.26; Cronbach’s ∝ = 0.905)**				
The range of activities is updated	0.754	4.22	0.86	0.604
The activity is pleasant	0.763	4.39	0.76	0.612
The tasks developed in the class are varied enough	0.740	4.43	0.71	0.712
The timetable is convenient for users	0.627	4.48	0.71	0.712
The activities end at the indicated time	0.692	4.50	0.66	0.601
You are informed about the benefits of this activity	0.668	4.45	0.68	0.528
You are satisfied with the quality/price ratio of the activity	0.657	4.42	0.72	0.427
I get the expected results from this activity	0.613	4.56	0.65	Minor 0.4
**Factor 4: communication (Mean = 4.38; Eigenvalue = 3.214; % Variance = 10,71; Cronbach’s ∝ = 0.864)**				
The facilities have some means to transmit your suggestions (suggestion box, bulletin board)	0.590	4.35	0.75	0.66
The information about the activities developed in the center is adequate	0.611	4.41	0.69	0.744
The range of activities is permanently updated	0.720	4.38	0.79	0.660
**Factor 5: facility staff (Mean = 4.55; Eigenvalue = 2.887; % Variance = 9.62; Cronbach’s ∝ = 0.827)**				
The service staff is available when required and is always willing to help you	0.635	4.46	0.65	0.686
The treatment of the facility staff is pleasant	0.572	4.63	0.55	0.753
There is good relationship between the service staff	0.559	4.60	0.58	0.770
It was easy to join the activity in which you participate	0.613	4.56	0.65	0.417

* N = 206; Likert scale = 1= least important /5 = most important; ^1^ Tests that show that the data obtained through the questionnaire were adequate to perform the factor analysis (requirements: Bartlett’s Sphericity Test χ^2^ (sig. > 0.05), KMO > 0.7 median, MSA = unacceptable for values below 0.5); ^2^ S.D: Standard deviation; Source: Authors’ own data.

**Table 2:** Satisfaction: Descriptive and factorial exploratory analysis.

Exploratory Factor Analysis ^1^; Cronbach’s ∝ = 0.909; χ^2^ (sig.): 1559.393 (0.000); KMO: 0.879; Measure of Simple Adequacy (MSA): (0.854−0.932) % Variance: 73.77	Item-Total Correlation	Mean*	S.D ^.2^	Loadings
**Factor 1: satisfaction with the facilities (Mean = 4.46; Eigenvalue = 3.404; %Variance = 28.36; Cronbach’s ∝ = 0.876)**				
Cleanliness	0.552	4.40	0.63	0.764
Dimensions of the different areas of the facilities	0.644	4.45	0.76	0.828
Accessibility	0.654	4.51	0.59	0.795
Ventilation	0.684	4.52	0.58	0.755
Cleanliness	0.609	4.45	0.74	0.761
**Factor 2: satisfaction with the organization of activities (Mean = 4.49; Eigenvalue = 2.821; % Variance = 23.50; Cronbach’s ∝ =0.885)**				
Hours in which they are developed	0.593	4.47	0.68	0.884
Use of time in the activity	0.641	4.49	0.62	0.812
Number of weekly hours dedicated to the activity	0.694	4.51	0.69	0.796
**Factor 3: satisfaction with the activity (Mean = 4.47; Eigenvalue = 2.628; % Variance = 21.89; Cronbach’s ∝ = 0.876)**				
The sessions are motivating	0.627	4.47	0.57	0.811
The intensity of the sessions is adequate	0.639	4.44	0.59	0.873
The sports equipment used is adequate	0.702	4.48	0.67	0.727
The duration of the sessions is adequate	0.705	4.51	0.58	0.663

* N = 206; Likert scale = 1 = least important /5 = most important; ^1^ Tests that show that the data obtained through the questionnaire were adequate to perform the factor analysis (requirements: Bartlett’s Sphericity Test χ^2^ (sig.> 0.05), KMO > 0.7 median, MSA = unacceptable for values below 0.5); ^2^ S.D: Standard deviation; Source: Authors’ own data.

**Table 3:** Measurement scale correlations of the perceived quality of service and user satisfaction.

	1	2	3	4	5	6	7	8	9	10	11
1. Gender	1.00										
2. Age	−0.009	1.00									
3. Monitor/coach	0.044	−0.070	1.00								
4. Facilities and material	0.073	0.024	0.521 *	1.00							
5. Activities	0.084	−0.054	0.631 *	0.755 *	1.00						
6. Communication	0.046	0.092 *	0.404 *	0.691 *	0.669 *	1.00					
7. Facility staff	0.152	0.048	0.572 *	0.636 *	0.657 *	0.490 *	1.00				
8. Satisfaction with the facilities	−0.098	−0.010	0.432 *	0.715 *	0.593 *	0.581 *	0.504 *	1.00			
9. Satisfaction with the organization of activities	−0.025	−0.044	0.508 *	0.625 *	0.710 *	0.576 *	0.583 *	0.453 *	1.00		
10. satisfaction with the activity	0.086	0.094	0.539 *	0.655 *	0.642 *	0.624 *	0.601 *	0.542 *	0.625 *	1.00	
11. Overall satisfaction	0.060	0.012	0.592 *	0.798 *	0.782 *	0.712 *	0.676 *	0.793 *	0.845 *	0.858 *	1.00

Note: * *p* < 0.001. Bilateral test; Source: Authors’ own data.

**Table 4:** Result of the regression analysis for the dimensions of the perceived quality of the service and satisfaction of the users.

	Dependent Variables			
	Overall Satisfaction	Satisfaction with the Facilities	Satisfaction with the Organization of Activities	Satisfaction with the Activity
**Control variables**				
Gender	−0.015	−0.037	−0.055	0.062
Age	−0.022	0.040	−0.106	0.021
**Independent variables**				
Monitor/coach	0.105 *	0.054	0.053	0.163 *
Facilities/material	0.325 **	0.540 **	0.087	0.198 *
Activities	0.214 **	0.015	0.399 **	0.095
Communication	0.225 **	0.160 *	0.149 **	0.260 **
Facility staff	0.162 **	0.037	0.180 **	0.186 **
**Model Information**				
R^2^	0.757	0.534	0.561	0.560
R^2^ corrected	0.748	0.518	0.545	0.545
F for Regression	88.149 **	32.437 **	36.099 **	36.046 **
Durbin-Watson Test	1.745	1.848	1.902	1.863

Note: Cell entries are standardized coefficients; * *p* < 0.05; ** *p* < 0.001. Bilateral test; Source: Authors’ own data.
